# Setting healthcare priorities in hospitals: a review of empirical studies

**DOI:** 10.1093/heapol/czu010

**Published:** 2014-03-05

**Authors:** Edwine W Barasa, Sassy Molyneux, Mike English, Susan Cleary

**Affiliations:** ^1^KEMRI Centre for Geographic Medicine Research – Coast, and Wellcome Trust Research Programme, P.O. Box 43640, Nairobi 00100, Kenya, ^2^Health Economics Unit, University of Cape Town, Observatory 7975, Cape Town, South Africa, ^3^Centre for Tropical Medicine, Nuffield Department of Medicine Research Building, University of Oxford, Old Road campus, Roosevelt Drive, Headington, Oxford, OX3 7FZ and ^4^Nuffield Department of Medicine & Department of Paediatrics, University of Oxford, Old Road Campus, Headington, Oxford OX3 7BN, Oxford, UK

**Keywords:** Priority setting, healthcare rationing, healthcare planning, hospitals

## Abstract

Priority setting research has focused on the macro (national) and micro (bedside) level, leaving the meso (institutional, hospital) level relatively neglected. This is surprising given the key role that hospitals play in the delivery of healthcare services and the large proportion of health systems resources that they absorb. To explore the factors that impact upon priority setting at the hospital level, we conducted a thematic review of empirical studies. A systematic search of PubMed, EBSCOHOST, Econlit databases and Google scholar was supplemented by a search of key websites and a manual search of relevant papers’ reference lists. A total of 24 papers were identified from developed and developing countries. We applied a policy analysis framework to examine and synthesize the findings of the selected papers. Findings suggest that priority setting practice in hospitals was influenced by (1) contextual factors such as decision space, resource availability, financing arrangements, availability and use of information, organizational culture and leadership, (2) priority setting processes that depend on the type of priority setting activity, (3) content factors such as priority setting criteria and (4) actors, their interests and power relations. We observe that there is need for studies to examine these issues and the interplay between them in greater depth and propose a conceptual framework that might be useful in examining priority setting practices in hospitals.

**KEY MESSAGES**

- There is a dearth of empirical work on hospital level priority setting practices and more so in smaller, rural hospitals in developing country contexts.
- The majority of empirical papers identified focused on hospital priority setting in larger, often referral hospitals in developed countries.
- Factors at play in hospital priority setting practices include (1) contextual factors such as decision space, resource availability, financing arrangements, availability and use of information, organizational culture and leadership, (2) priority setting processes, (3) content factors such as such as priority setting criteria and (4) actors, their interests and power relations.
- Research that aims to examine priority setting practices in hospitals would benefit from applying a health policy lens to their analysis.

## Introduction

Priority setting refers to the distribution of resources among competing programmes and patients or patient groups (McKneally *et al*. 1997). Given that healthcare demand outstrips available resources, the judicial use of resources through appropriate priority setting has been considered a key determinant of health system performance (Martin 2007). For example, it has been shown that a reallocation of 50% of the health budget from interventions that are less cost effective to those that are more cost effective could result in a 64% increase in years of life saved in the East African region (Bobadilla *et al.* 1994). In addition, the ‘Tanzania Essential Health Intervention Project’ suggested that targeted investments guided by proper prioritization resulted in a 40% reduction in child mortality in test districts (De Savigny *et al*. 2004).

Whereas priority setting occurs in every health system, research has mainly focused on macro and micro level processes and rarely on the meso level, particularly hospitals (Martin *et al*. 2003a). This is perhaps surprising given the critical role that hospitals play in the delivery of healthcare services and the observation that they absorb a significant portion of healthcare resources. For example, it has been observed that public hospitals absorb 30–50% of government budgetary allocations to the health sector in both developed and developing countries (Mills 1990; Martin *et al*. 2003a). Understanding how these hospitals set their priorities and the factors that influence their allocation of resources is therefore imperative. We conducted a thematic review of empirical literature on priority setting at the hospital (meso) level with the aim of describing what is known about priority setting practices and exploring the factors that influence this practice.

## Methods

### Literature search

We carried out a literature search in December 2012 in PubMed, EBSCOHOST, Econlit databases, Google scholar and websites of the World Health Organization, the World Bank, Management Science for Health, US Agency for International Development and the Organization for Economic Cooperation and Development. In a first step, we performed a search using the following keywords: ‘hospital’ and ‘priority setting’ or ‘rationing’ or ‘healthcare rationing’ or ‘planning’ or ‘decision making’ or ‘strategic planning’ or ‘resource allocation’ or ‘health technology assessment’ or ‘budgeting’. Reference lists of selected papers were also manually searched for relevant papers. We limited the search to studies published in the English language that were available from January 1990 to December 2012. Next, we only included studies in the review if they reported empirical data on priority setting practice in hospitals. In this step, we initially screened study abstracts using these criteria and subsequently obtained full-text formats for studies deemed relevant. The final inclusion of studies in the review was based upon a detailed assessment of the full-text formats (studies for which no full-text format was available were excluded). All abstracts and full-text formats were reviewed independently by two authors. We then classified studies according to five general characteristics: (1) country (ies) where the studies were conducted, (2) study design, (3) priority setting activity and (4) study objectives.

### Analysis of selected papers

First, we read through the selected papers to familiarize ourselves with the studies and identify key ideas and themes. Drawing on the Walt and Gilson policy analysis framework that focuses on four key domains (content, context, process and actors) (Walt and Gilson 1994), we identified themes and concepts that clustered around each of these main domains. Each of the selected papers was imported to NVIVO version 10 software (QSR International) and coded using this thematic framework. Data were then lifted from their original context and rearranged according to the appropriate thematic reference and summarized in charts. Finally, a synthesis and interpretation of each theme and interrelationships between themes was conducted.

## Results

The first step in the literature search resulted in a total of 2659 papers. In total, 2531 studies were excluded on the basis of their title. The abstracts of the remaining 136 studies were assessed, and a further 93 papers excluded. Three more papers were excluded because they were not available online. An assessment of the full-text formats of the remaining 40 papers resulted in a further 16 exclusions. A total of 24 studies were finally included in the review (Table 1). This section will first present the characteristics of selected studies, their objectives and methodological approaches. In line with the policy analysis framework employed in the review, this will then be followed by findings on the content, context, process and actors of priority setting processes. Finally, findings on how priority setting processes were evaluated in the studies are presented. Further information on these result areas for each of the papers selected for the review is presented in Supplementary data.

**Table 1:** General characteristics of the 24 included empirical hospital priority-setting studies

Study	Country	Study design	Study setting	Priority setting activity	Study objectives
Reeleeder *et al.* (2006)	Canada	Qualitative cross-sectional study	Forty-six hospital in Ontario, Canada	Allocation of hospital resources (and budgets) between departments and service areas	To describe the role of leadership in health services priority setting from the perspective of hospital leaders, and provide a set of lessons for effective priority setting practices in healthcare facilities.
Gibson *et al.* (2005)	Canada	Qualitative case study	A tertiary-care teaching hospital with 612 acute-care beds, 543 long-term care beds, 74 nursery beds and 22 rehabilitation beds	Allocation of hospital resources (and budgets) between departments and service areas—hospital strategic planning process	To examine power differences associated with institutional roles in the context of management decision making about organizational priorities.
Bochner *et al.* (1994)	Australia	Qualitative case study	A tertiary referral hospital with about 900 beds	Health technology acquisition—medicines formulary management	To report experiences and initial responses from the hospital staff to a method to assign ranking priorities by means of a formal scoring system used for previously unfunded initiatives to allow their serial and orderly introduction into the hospital formulary.
Vissers (1995)	Denmark	Mixed methods case study	A hospital in Denmark	Health technology acquisition	To develop a model for resource allocation based on patient flow and to test this model on the allocation of hospital resources.
Durand-Zaleski (1996)	France	Interventional case study	A hospital in France	Health technology acquisition	To describe the testing of a tool to help decision makers establish priorities among medical projects by scoring and ranking projects.
Madden *et al.* (2005)	Canada	Qualitative case study	A network of three teaching hospitals in Toronto, Canada	Allocation of hospital resources (and budgets) between departments and service areas—clinical activity target setting process	To describe priority setting in a hospital and evaluate it using ‘accountability for reasonableness’, with particular attention to the appeal process.
Martin *et al.* (2003b)	Canada	Qualitative case study	A tertiary-care teaching hospital with 612 acute-care beds, 543 long-term care beds, 74 nursery beds and 22 rehabilitation beds	Allocation of hospital resources (and budgets) between departments and service areas—hospital strategic planning process	To describe priority setting in the context of a hospital strategic planning initiative and to evaluate using ‘accountability for reasonableness’.
Martin *et al.* (2003a,b)	Canada	Qualitative case study	A network of three teaching hospitals in Toronto, Canada	Health technology acquisition—medicines formulary management	To describe priority setting for new drugs in a hospital and to evaluate this process using ‘accountability for reasonableness’.
Rosenstein *et al.* (2003)	USA	Quantitative survey	Nineteen hospitals in the USA	Health technology acquisition	To describe the structure and processes used by Veterans Health Administration (VHA) west coast hospitals to perform new technology assessments.
Bell *et al.* (2004)	Canada	Qualitative case study	A large tertiary hospital in Toronto, Canada	Allocation of hospital resources (and budgets) between departments and service areas—priority setting during a disease outbreak Severe Acute Respiratory Syndrome (SARS)	To describe and evaluate priority setting in a hospital in response to SARS.
Reeleeder *et al.* (2005)	Canada	Quantitative survey	Forty-six hospital in Ontario, Canada	Allocation of hospital resources (and budgets) between departments and service areas	To elicit hospital decision makers’ self-report of the fairness of priority setting in their hospitals using ‘accountability for reasonableness’.
Greenberg *et al.* (2005)	Israel	Quantitative survey	Twenty-six acute care hospitals in Israel	Health technology acquisition	To explore the decision-making process in adopting new technologies at the hospital level.
Kapiriri and Martin (2006)	Uganda	Qualitative case study	A referral hospital with 1500 patient beds	Allocation of hospital resources (and budgets) between departments and service areas	To describe priority setting in a Ugandan hospital and to evaluate the description using the ethical framework, AFR.
Sharma *et al.* (2006)	Canada	Qualitative case study	A community hospital with 425 patient beds	Health technology acquisition—adoption of advanced laparoscopic surgery	To describe the current decision-making processes for the adoption of advanced laparoscopic surgery at a community hospital in Toronto, Canada and to analyse the decision-making process using the ethical framework AFR.
Ehlers *et al.* (2006)	Denmark	Quantitative survey	Thirty-three hospitals in Denmark	Health technology acquisition	To evaluate local decision support tools used in the Danish hospital sector from a theoretical and an empirical point of view.
Kapiriri *et al.* (2007)	Uganda, Canada, Norway	Qualitative case study	Three hospitals, one in Uganda, one in Canada and the other in Norway	Allocation of hospital resources (and budgets) between departments and service areas	To describe the process of healthcare priority setting in Ontario, Canada, Norway and Uganda at the macro, meso and micro levels and to evaluate the description using AFR and to identify lessons of good practice.
Danjoux *et al.* (2007)	Canada	Qualitative case study	An urban university academic health sciences centre with ∼500 patient beds	Health technology acquisition—endovascular aneurysm repair	To describe and evaluate the decision-making process for the adoption of a new technology for repair of abdominal aortic aneurysms-endovascular aneurysm repair.
Gallego *et al.* (2007)	Australia	Qualitative case study	A 300-bed university-affiliated, tertiary acute care hospital	Health technology acquisition—medicines formulary management	To describe the operations of the first reported High Cost Drug Sub-Committee in a public hospital in Australia and to evaluate the decision-making process using the ethical framework of AFR.
Haselkorn *et al.* (2007)	USA	Quantitative survey	Twenty-seven hospitals in the USA	Health technology acquisition	To assess the structure, processes and cultural support behind hospital committees for new technology planning and approval.
Gordon *et al.* (2009)	Argentina	Qualitative case study	An acute care tertiary level hospital with 350 beds	Allocation of hospital resources (and budgets) between departments and service areas	To describe priority setting in an acute care municipal level public hospital in Buenos Aires and to evaluate the priority setting process using an ethical framework for fair processes.
Valdebenito *et al.* (2009)	Chile	Qualitative case study	A 600 bed referral and teaching hospital in Chile	Allocation of hospital resources (and budgets) between departments and service areas—resource allocation do departments and services in the hospital	To describe, using qualitative case study methods, and evaluate, using the ethical framework ‘accountability for reasonableness’, priority setting in a hospital in Chile.
Mitchell *et al.* (2010)	USA	Qualitative case study	Four hospitals in the USA	Health technology acquisition	To describe two evidence reports from the hospital-based Health Technology Assessment (HTA) centre which required the integration of local data.
Govender *et al.* (2011)	South Africa	Quantitative survey	Twenty-one hospital managers in South Africa, number of hospitals not specified	Health technology acquisition	To adapt and use the Danish Center for Health Technology Assessment (DACEHTA) mini-HTA tool to assess past decisions made by South African hospital managers, as applied to selected medical devices.
Astley and Wake-Dyster (2001)	Australia	Qualitative case study	A division of women's and children’s hospital in Adelaide, Australia	Allocation of hospital resources (and budgets) between departments and service areas—reallocate hospital resources to maximize health outcomes by developing a new hospital service profile	To describe priority setting and resource allocation undertaken by a division of the women’s and children’s hospital, in Adelaide.

### Characteristics of selected studies

Of the 24 papers, 20 were focused on developed country experiences, while only 5 included developing country contexts. One of the papers reported a multi-country study that compared priority setting practices in two developed country hospitals (Canada and Norway) and one developing country hospital (Uganda). Ten studies were conducted in Canada, three each in Australia and the USA, two in Denmark and Uganda and one each in Argentina, Chile, Norway, Israel, France and South Africa.

Of the selected papers, 15 included tertiary level hospitals, 13 of which were also teaching hospitals, while 1 study was conducted in a community hospital. The level and type of hospital in the remaining eight studies was not clear. Fourteen studies were conducted in public hospitals, two in faith owned hospitals and one in a network of private hospitals. Seven studies were not clear about the ownership of the hospitals where the study was conducted.

### Objectives and methodological approaches of selected papers

Of the 24 papers, only 2 sought to introduce a priority setting method, while 22 sought to describe and/or evaluate the existing priority setting process. Of the latter, 13 sought to describe and evaluate the priority setting process, 7 sought only to describe the priority setting process and 2 sought only to evaluate the priority setting process. Thirteen of the evaluation studies employed the ethical framework accountability for reasonableness (AFR) (Daniels 2000), while one evaluated health technology assessment using an adapted mini-health technology assessment tool and another using no specific framework or tool.

The allocation of hospital resources and budgets to departments and service areas within the hospital was examined in 11 of the selected studies, while the remaining 13 specifically examined health technology assessment in hospitals. Of these, three looked at the medicines formulary management process, two looked at acquisition of surgical technology, while the remaining eight looked at the technology acquisition process in general.

Most papers (*n* = 18) employed case study methodology, while six employed quantitative survey methodology. Two of the 18 case studies were interventional, while the rest were descriptive explanatory, and all were qualitative, with the exception of one mixed method case study.

### Content of priority setting

#### Criteria used in priority setting

Formal and informal criteria were used to set priorities. Formal criteria are objective criteria that, at least on paper, hospitals claim to use in priority setting. These could be classified as health criteria, economic criteria and administrative criteria, as illustrated below. Informal criteria refer to subjective considerations that influence priority setting practices in hospitals.

*Formal criteria. *In allocating budgets to departments and health services, the main health criteria used were the perceived medical need in the hospital’s catchment area. For example, a study in a referral hospital in Uganda showed that disease prevalence in the hospital’s catchment area was considered in making decisions about what services to offer (Kapiriri and Norheim 2004). Burden of disease was also an important criteria in priority setting in hospitals in Canada (Kapiriri *et al*. 2007), Norway (Kapiriri *et al*. 2007), Chile (Valdebenito *et al*. 2009) and Argentina (Gordon *et al*. 2009). The rule of rescue also featured prominently whereby emergencies received high priority (Kapiriri *et al*. 2007). For health technology assessments and medicines selection, medical criteria included effectiveness, safety, ease of use and capacity of staff to employ the technology, patient benefits in terms of health outcomes and the nature of the technology/medicines. The latter was described in terms of whether it was a proven, new or investigational therapy. Proven therapies were often preferred.

Administrative criteria included strategic alignment and alignment with regional/national priorities, policies and objectives. Examples were found in both developed (such us Canada, Norway and Australia) and developing country (such as Uganda and Argentina) hospitals (Kapiriri *et al*. 2007; Gordon *et al*. 2009). Priority setting in hospitals in developed countries was also guided by organizational strategies, goals and vision (Martin *et al.* 2003a; Kapiriri *et al*. 2007). For example, a study of priority setting in three teaching hospitals in Canada showed that decisions were made based on local strategic fit, and academic commitment and research focus (Gibson *et al*. 2004). Hospitals also seemed to favour innovation in health technologies providing perceived competitive advantage over other hospitals.

Economic criteria included historical budgeting, revenue generating potential, budget impact and costs to patients. Cost effectiveness was a criterion considered in only two studies. Consideration was, however, given to whether the new interventions were affordable (Madden *et al.* 2005; Kapiriri *et al*. 2007; Valdebenito *et al*. 2009).

*Informal criteria. *Informally, personal relationships and mutual benefit, lobbying, level of ambition and bargaining ability of departmental heads and political interests among actors often dominated priority setting decisions especially in developing countries (Kapiriri and Norheim 2004; Gordon *et al*. 2009). For example, in a hospital in Argentina, it was reported that allocations depended on whether the hospital managers and departmental heads enjoyed good relations and the potential for mutual benefit between them (Gordon *et al*. 2009). In addition, given that decision making was centralized, priorities were aligned to meet the political goals of local politicians rather than the health needs of the population (Gordon *et al*. 2009). In Uganda, even though the formal criteria of need determined that the paediatric department, which received almost 40% of the hospital emergencies, is given higher priority, the surgical department was given greater priority because of its perceived prestige, and because it had managers who were better at ‘lobbying, making noise and quickly use up their resources’ (Kapiriri and Martin 2006).

### Context of priority setting

#### Decision space

Decision space refers to the range of effective choices or discretion that local authorities or institutions are allowed by central authorities (Bossert 1998; Bossert and Beauvais 2002). This space can be formal (as defined by policies and regulations) and informal (choices exercised in practice but not formally defined) (Bossert 1998; Bossert and Beauvais 2002). The decision space for hospital level priority setting was influenced by the structure of the health system and the nature of the priority setting activity. For example, in countries such as Canada and Norway where the health system was significantly decentralized, hospitals had greater decision-making latitude (Kapiriri *et al*. 2007), while in Chile, a country with a less decentralized health system, priority setting at the hospital level was predominantly guided by national decisions with little discretion at the hospital level (Valdebenito *et al*. 2009). Hospitals generally had most discretion over decisions about medicines formularies and adopting new technologies compared to decisions about choice of programmes and allocations across programmes and departments.

#### Resource gap

The reality of constrained resources compelled decision makers to tackle the issue of healthcare rationing (Bochner *et al*. 1994; Martin *et al*. 2003a,b; Gallego *et al*. 2007). In Australia, for example, shrinking healthcare resources resulted in vigorous debate about the need for, ethics of and possible methods for cost containment and rationing of health services (Gallego *et al*. 2007). Increasing demand and reduced revenues also influenced the financing arrangements in hospitals. In Uganda, for example, an increasing budget deficit led to the capping of budgets and introduction of line budgeting which reduced the flexibility of priority setting (Kapiriri and Martin 2006). Budget caps for new medicines were also implemented in an Australian hospital to contain costs in the face of reducing resources (Bochner *et al*. 1994).

#### Financing arrangements

Hospital financing arrangements also played a key role in determining priority setting practices in hospitals. This influence appeared to be in two forms (1) through the conditions associated with the financing sources and (2) through the incentives engendered by financing arrangements. For example, given that Chile has a mixed publicly and privately financed healthcare system, hospitals were required to employ guidelines that aligned their priorities to those prescribed by both systems (Valdebenito *et al*. 2009). Funding arrangements also generated incentives that influenced priority setting practice. Hospitals which were funded by a global budget were less willing to fund incremental use of new technology compared to hospitals funded under different models, such as fee for service (Sharma *et al*. 2006; Danjoux *et al*. 2007). Operating under line budgets reduced the flexibility of hospitals in choosing priorities and allocating resources across them (Kapiriri and Martin 2006). The introduction of budget caps also discouraged the adoption of new technologies as it required cutting allocations to hospital services (Sharma *et al*. 2006).

#### Organizational culture

Two important aspects of culture seemed crucial enablers of systematic priority setting processes, namely the importance attached to the use of evidence and the openness to consultative and deliberative processes (Astley and Wake-Dyster 2001; Madden *et al*. 2005; Danjoux *et al*. 2007). For example, in Chile, a country with a history of dictatorship and military rule, a government culture that discourages disagreement impeded the implementation of an appeals and revisions process (Valdebenito *et al*. 2009). Specifically for technology adoption, cultural drivers for technology assessment and acquisition included a proactive approach to seeking new technology, having an organizational commitment to innovation and placing high importance on integration of technology planning with the mission and strategic plan of the organization (Astley and Wake-Dyster 2001; Danjoux *et al.* 2007; Haselkorn *et al.* 2007).

#### Leadership

Within hospitals, leadership emerged as one of the key factors influencing the process of priority setting. A study on the role of leadership in priority setting reported that leaders are expected to foster goals and a vision for the hospital; create alignment between goals, vision, resources and skills, actors and processes; develop and maintain relationships among actors; embody and promote desired values and establish an effective process for priority setting (Reeleder *et al*. 2006). The commitment of hospital leaders to implementing a fair and legitimate process was considered crucial to meeting the conditions of the ethical priority setting framework, AFR (Bell *et al*. 2004; Madden *et al*. 2005; Reeleder *et al*. 2005; Kapiriri and Martin 2006; Kapiriri *et al.* 2007; Gordon *et al.* 2009, Valdebenito *et al*. 2009). Within this framework the role of leadership seems to hinge on two points. First, the enforcement condition of A4R suggests that good leadership involves attention to the ethical aspects of priority setting. Second, leadership approaches describe a variety of values and behaviours which align with, and can be viewed as enablers for, A4R.

### Process of priority setting

**Figure 1:**
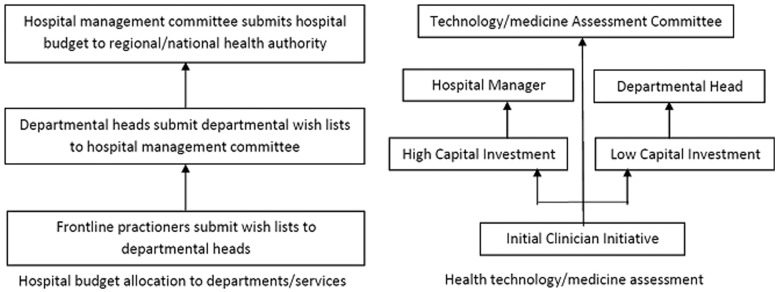
Hospital priority setting processes.

The process of priority setting in hospitals was dependant on the priority setting activity (Figure 1). For hospital budget allocations to departments and service areas, at least on paper, the priority setting process began with frontline staff (clinical and non-clinical staff within all the departments of the hospital) submitting their wish lists to their departmental heads (Kapiriri and Martin 2006; Kapiriri *et al*. 2007; Gordon *et al*. 2009). In practice, the departmental heads compiled departmental wish lists and submitted them to the hospital management without consulting frontline staff (Kapiriri and Martin 2006; Valdebenito *et al*. 2009). Departmental priorities were compiled to form hospital priorities by a hospital management committee whose membership comprised of all or some of the departmental heads and the executive hospital management. These hospital priorities/budget allocations were thereafter submitted to a hospital management board, whose membership included external stakeholders such as the community, for approval (Kapiriri and Martin 2006; Kapiriri *et al*. 2007; Gordon *et al*. 2009). Thereafter, plans were submitted to the regional or national health authorities/ministries for final approval.

Decision making for new technologies and medicines often began with clinician interest and initiative (Bochner *et al*. 1994; Martin *et al*. 2003b; Sharma *et al.* 2006; Danjoux *et al.* 2007; Gallego *et al*. 2007). Suggestions for new technologies and medicines were thereafter processed through three possible channels (Figure 1). For medicines, often these suggestions were presented to an assessment committee which employed selection criteria to make decisions about their selection and inclusion in the hospital formulary (Bochner *et al*. 1994; Martin *et al.* 2003b; Gallego *et al.* 2007). This committee goes under different names such as the medicines and therapeutics committee, or pharmacy and therapeutics committee. For other technology such as surgical technology, decision making depended on the level of capital investment required (Sharma *et al.* 2006; Danjoux *et al*. 2007). For technology that required a low capital investment, decision making for adoption was made by departmental heads. When a proposed technology was associated with significant capital investment, final adoption decisions were made by the hospital manager/Chief Executive Officer (Greenberg *et al*. 2005). In some hospitals, technology assessment committees had the responsibility of evaluating and making decisions about the adoption of new technologies (Greenberg *et al*. 2005).

#### Availability and use of information

The availability and quality of information for decision making had a significant influence on priority setting practice. The lack of information was the most frequent priority setting obstacle identified by the studies in the review. Hospital decision makers generally lacked sufficient and reliable information for decision making (Gordon *et al*. 2009; Greenberg *et al*. 2009). The absence of quality data provided loopholes for the use of informal/subjective considerations in the priority setting process (Gordon *et al*. 2009). Lack of information also resulted in assessments being conducted after technologies had been adopted and widely used (Greenberg *et al*. 2009). Decision makers felt that the availability of quality information would improve the priority setting process (Martin *et al*. 2003a,b; Madden *et al.* 2005; Reeleder *et al*. 2005).

### Actors, their power and interests

Whereas the different actors and their influence permeate through all the other themes, we discuss here some specific observations. Actors (stakeholders) in the priority setting process included national and regional health policy makers and planners, local politicians, donor organizations, community members, patients, hospital administrators/executives, hospital department heads and frontline practitioners (non-managerial clinical and non-clinical staff working directly with clients). The involvement of national and regional health policy makers was dependent on where the policy making authority was vested. In high-income countries such as Canada, where regional health authorities made policy, hospitals aligned their priorities with those of the regional health authorities (Kapiriri *et al*. 2007). In low- and middle-income countries such as Uganda and Chile, where policy making was done at the national level, the hospital priorities were aligned with national priorities (Kapiriri and Martin 2006; Valdebenito *et al*. 2009). Donor organizations influenced decision making in Uganda, a developing country setting, where resource scarcity was extreme (Kapiriri and Martin 2006).

Community involvement was in theory effected through representation in hospital management boards. In one study, community and patient involvement was effected through surveys of community and patient views (Astley and Wake-Dyster 2001). The minimal involvement of the community and patients was attributed to, among others, the perception that the community and patients lack understanding of medical issues and would represent a biased opinion by solely arguing for the merit of the particular interventions for which they were concerned (Martin *et al*. 2003b). Within the hospital, priority setting was dominated by hospital administrators/managers, with some settings reporting minimal involvement of frontline practitioners (Kapiriri and Martin 2006; Kapiriri *et al.* 2007). Reasons for the minimal involvement of practitioners included time constraints, and lack of interest (Kapiriri and Martin 2006). Power struggles between practitioners and managers who were reluctant to share decision-making power, and frustration by practitioners when their concerns were not addressed, also contributed to the non-participation of practitioners (Kapiriri and Martin 2006).

Other than the range of stakeholders involved, the power differences between these stakeholders had a major influence in the priority setting process in hospitals (Gibson *et al*. 2005). Power differences exist when some actors in the priority setting process have the capacity to influence priority setting outcomes more than others. This occurs given that hospital decision-making environments tend to be hierarchical and politically complex (Gibson *et al*. 2005). Power was derived from several sources. For example, actors with control over the budget had more power and hence influence over priority setting decisions (Gordon *et al*. 2009). The senior hospital managers exercised more power over decisions compared with other hospital managers and frontline practitioners by virtue of their position as senior managers (Gibson *et al*. 2005). For example, the hospital executive in a hospital in Argentina indicated that they did not need to consult the hospital management committee when requesting additional staff allocations (Gordon *et al*. 2009). A study of a hospital in Uganda reported power struggles between management and frontline workers, with managers reluctant to share decision-making responsibility. Actor power derived from possession of specialized skills and certain personal characteristics were also exercised (Gibson *et al*. 2005). For example, a study of decision making for a new surgical technology in Canada reported conflict between surgeons and radiologists over leadership of the process. There was also conflict between professional groups in hospitals (Astley and Wake-Dyster 2001; Haselkorn *et al*. 2007) leading to competitive and defensive rather than collaborative behaviour. A study in Canada reported that actors with greater persuasive skills had greater power to influence the planning process (Gibson *et al*. 2005).

Different actors often had varying values and hence depending on the power they possess, influence priority setting in line with their values. For example, two decision-making systems were in conflict in hospitals namely the ‘medical-individualistic’ decision system and the ‘fiscal-managerial’ decision system (Greer 1985). While clinicians, who subscribe to the ‘medical-individualistic’ decision system, were concerned with individual patient outcomes, administrators/managers, who subscribe to the ‘fiscal-managerial’ decision system, were concerned with the implications of decisions on the budget (Danjoux *et al*. 2007; Gordon *et al*. 2009). This conflict was more evident in scenarios where decisions affected identifiable patients such as medicines selection processes (Gallego *et al*. 2007).

### Evaluation of priority setting process

The dominant priority setting framework used in evaluation was AFR. AFR proposes that a legitimate and fair priority setting process should meet the following four conditions: (1) publicity, (2) relevance, (3) revisions and (4) enforcement (Daniels and Sabin 2002).

All the studies that evaluated priority setting using the AFR framework reported that the relevance condition was not met. While there were some formal reasons, often informal reasons were also considered. These included political reasons and actor interests, persuasion skills of actors and relationships between the actors. The lack of evidence or information to support formal reasons is also seen as a barrier by decision makers. Given that frontline staff and the public were often excluded, not all the relevant actors participated in the process. It was generally observed that the publicity condition was not met because while decisions were communicated, this communication was often only for the people that took part in decision making, thereby excluding other stakeholders such as frontline workers and/or the public. In addition, reasons/rationales for the decisions were not communicated. Of the 12 studies, only 4 reported the presence of a formal appeals mechanism, while the other 8 reported the presence of informal mechanisms where dissatisfied staff would seek redress directly with the hospital chief executive. There was generally no mechanism for enforcement of a fair and legitimate process other than in four studies.

## Discussion

We set out to review empirical studies of meso level priority setting in hospitals. To our knowledge this is the first review of empirical studies on hospital level priority setting. Our review confirms that there is limited research attention given to priority setting at this level. Given that hospitals consume a significant proportion of health system resources, and act as avenues for delivery of key healthcare interventions, understanding how and where they put their resources is an important research and practice question. Another key observation is that most studies of priority setting in hospitals focused on developed country settings with few being conducted in developing countries. Most of the studies were conducted in tertiary, often teaching hospitals. Such hospitals are relatively large and act as referral hospitals. These hospitals are often semi-autonomous institutions whose management structures, operations, resources and target users are very different from lower level hospitals. There is, therefore, a gap in understanding how smaller, non-referral hospitals set their priorities and allocate their resources.

Most of the studies in this review employed qualitative case study methodology. The use of this approach allowed for an in-depth exploration of not just the what, but how and why of priority setting practices in these hospitals. Where case study methodology was not employed, quantitative surveys were used. These surveys were often limited to reporting frequencies against selected characteristics of priority setting and were unable to explore how and why the different aspects of priority setting interact and affect each other. There is perhaps a need to develop and employ mixed method approaches in the study of priority setting. For example, while qualitative methods were particularly good at eliciting the criteria used in setting priorities, they were unable to determine the relative importance of these criteria. Application of priority setting criteria implies trade-offs between competing criteria. Determining the relative importance of priority setting criteria is, therefore, an important ingredient in systematic priority setting processes. There are a number of quantitative methodologies that can be employed to elicit preferences for priority setting criteria, ranging from simple rating and ranking scales, self-explication methodology and choice experiments, to more complex discrete choice experiments (Ryan *et al.* 2001; Pavlova *et al*. 2003; Baltussen and Niessen 2006).

All but one of the studies that sought to evaluate priority setting employed the AFR framework. This is an ethical framework developed by Daniels and Sabin that aims to be ‘a practical, yet theoretically defensible, account of how societies should set limits to and priorities for health care’ (Eddy 1991). The framework argues for a focus on the ‘process’, rather than the ‘outcome’ of priority setting, given the lack of consensus about universal criteria. AFR proposes that the goal of priority setting should be fairness and legitimacy. The studies captured in this review, therefore, endeavoured to evaluate priority setting processes against the four conditions of AFR such as relevance, publicity, appeals and revision and enforcement. The ‘publicity’ criterion holds that resource allocation decisions must be public, including the grounds for making them. The criteria for ‘relevance’ require that the basis on which allocative decisions are made must be ones that ‘fair-minded people can agree are relevant to meeting the healthcare needs fairly under reasonable constraint’ (Daniels and Sabin 1997). Arguments should rest on scientific evidence, though not necessarily a specific kind of evidence (Daniels and Sabin 1998). The ‘revisions and appeals’ process criterion requires that there is an institutional mechanism that provides for channels for appeals to decisions and subsequent revisions of decisions in light of further arguments. The ‘enforcement’ criterion requires that some form of regulation exists to make sure that the first three conditions are met (Daniels 2008). While a focus on the fairness and legitimacy of priority setting processes is indisputably important, understanding what substantive principles are employed in priority setting and how they are operationalized in local context is equally important. For example, do hospitals allocate their resources across services according to severity of disease or efficiency? And how do they define and specify these principles? While this distinction between substantive and procedural approaches to priority setting suggests that the two are incompatible, there is increasing consensus on the need to combine these approaches (Ham and Coulter 2000; Ruger 2004; Kamm 2005). There is, therefore, a need to develop approaches that combine the use of substantive principles that are widely accepted with fair processes.

Most of the studies were inspired by a framework proposed by Martin and Singer (2003) which recommends a strategy for improving priority setting that involves (1) describing priority setting in the context where it occurs, (2) evaluating the description using an ethical framework and (3) improving priority setting based on the evaluation. In elaborating this approach, they argue that any sustainable strategy to improve priority setting must be built on a continuous learning platform that, at the very least, captures how priority setting decisions are actually made (Martin and Singer 2003). This, they argue would necessitate a description of the priority setting contexts, processes and actors involved. Our review highlights a range of factors influencing priority setting in these institutions regarding context (e.g. financing arrangements, leadership, organizational culture, level of resourcing and demand for health care), process (e.g. procedures and tools used) and content (e.g. guidelines and criteria of priority setting) as well as the importance of the interests and influence of the key actors involved in the process. Some critical aspects of priority setting appear to have been neglected by the studies reviewed, however. For example, while contextual issues such as financing arrangements and decision-making capacity of managers are arguably important in priority setting processes, these were at best minimally explored. Also given that priority setting is a social process with a range of actors, the power relationships between these actors and how these influence the process warrant a more in-depth examination.

**Figure 2:**
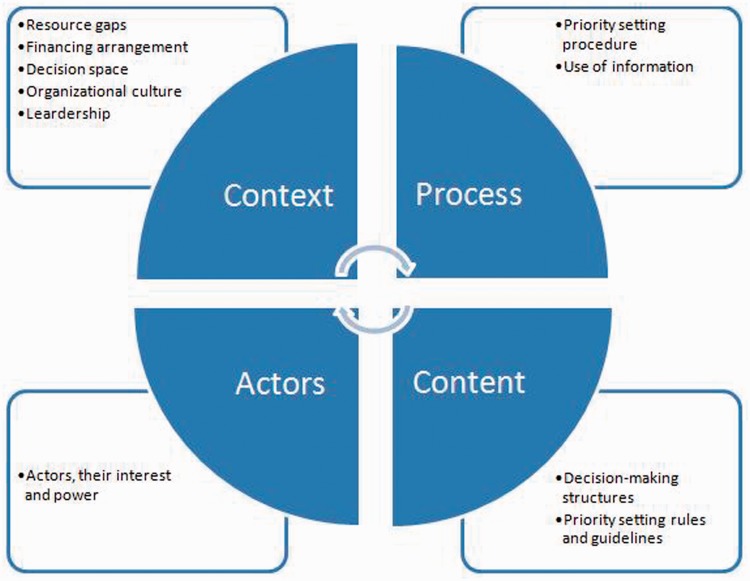
Framework for examining priority setting practice in hospitals.

Given the difficulty in comparing and analysing studies with different objectives, approaches and methods, we found it useful to apply an a priori framework (Walt and Gilson policy analysis framework) to examine the selected studies. This made it easier to determine what to look for in the papers, organize the extracted data and structure the synthesis of findings. On the basis of the review and drawing on policy analysis frameworks (Walt and Gilson 1994; Buse 2007; Gilson and Raphaely 2008), we propose that studies that aim to analyse priority setting practices use a health policy lens. Future research that aims to examine priority setting would benefit from carefully considering four interrelated areas (Figure 2):

- Context: What contextual issues influence the priority setting processes? This would include issues such as health system structure, political arrangements, financial and economic factors, capacity of decision makers, nature and level of demand for healthcare services, decision space and organizational culture.
- Content: What priority setting guidelines are in place, and what criteria are used to allocate resources?
- Process: What are the procedures and tools hospitals should use to set priorities? Are these procedures and tools used? If not why?
- Actors: Who are the relevant internal and external actors involved in the priority setting process? What are their roles, interests, level of influence and power relations? How does this influence priority setting practice?

## Conclusion

Hospitals are a major sector within most health systems consuming considerable resources. There is, however, a dearth of literature on priority setting in hospitals in developing countries and in particular little attention has been paid to lower level hospitals as opposed to larger, referral hospitals. Most priority setting studies employed a qualitative case study methodology, a suitable approach for examining complex social phenomena that are often highly embedded in context. However, broader approaches that include mixed methods should also be considered. Our review identified a range of factors that affect priority setting practice in hospitals. These factors provide potential policy levers that could be used to influence priority setting processes. We have also proposed a framework that, in our view, could be useful in examining priority setting processes and potentially informing the design of system interventions to influence priority setting at the meso level in hospitals. In increasingly devolved systems greater attention to the practice and consequences of priority setting are required to promote accountability, efficiency, effectiveness and equity.

## Supplementary Data

Supplementary data are available at *HEAPOL* online.

Supplementary Data

Translated Abstracts
