# Supplementary material for: Setting healthcare priorities in hospitals: a review of empirical studies
Source: Health Policy Plan. 2014 Mar 5;30(3):386–96. doi: 10.1093/heapol/czu010 (PMC4353893; doi:10.1093/heapol/czu010)
Supplement: Translated Abstracts [file supp_30_3_386_v2_index.html]

Setting healthcare priorities in hospitals: a review of empirical studies — Translated Abstracts 

# Setting healthcare priorities in hospitals: a review of empirical studies

## Translated Abstracts

files

**Files in this Data Supplement:**

- Chinese Abstract - pdf file
- French Abstract - pdf file
- Spanish Abstract - pdf file
