# Supplementary material for: Setting healthcare priorities in hospitals: a review of empirical studies
Source: Health Policy Plan. 2014 Mar 5;30(3):386–96. doi: 10.1093/heapol/czu010 (PMC4353893; doi:10.1093/heapol/czu010)
Supplement: Translated Abstracts [file supp_czu010_czu010_Chinese.pdf]

医院中的医疗优先级设立：对现有实证研究的综述

现有的关于医疗优先级的设立的研究主要集中在宏观（全国性）和微观（临床）层面，对中间层面（机构、医院）的研究在一定程度上被忽视了。考虑到医院在提供医院服务中扮演的角色以及医院吸收了医疗系统很大比例的资源，对其研究的忽视实在令人惊讶。我们对现有的实证研究进行了一项针对此论题的文献研究，以期找出影响医院层面优先级设立的因素。对 PubMed，EBSCOHOST，Econlit 数据库以及谷歌学术搜索也进行了系统性的研究，通过对这些关键网站以及相关论文引用目录的人工搜索来对本研究进行补充。我们从发达和发展中国家中找出了 24 篇论文。通过一个政策分析框架来检查并综合这些论文的研究发现。研究得出，医院的优先级设立主要受以下几个因素的影响：（1）情境因素，比如决策空间，可用资源，资金安排，信息的可利用性和使用，组织文化与领导；（2）不同优先级活动引起的优先级设立过程；（3）内容因素，比如优先级设立的标准；（4）参与者，这些参与者的利益以及能力关系。我们观察得出，应该对这些方面以及它们之间的关系与互动进行更深入的研究，同时建立一个概念框架对研究医院层面的优先级设立也是有帮助的。
