# Supplementary material for: Setting healthcare priorities in hospitals: a review of empirical studies
Source: Health Policy Plan. 2014 Mar 5;30(3):386–96. doi: 10.1093/heapol/czu010 (PMC4353893; doi:10.1093/heapol/czu010)
Supplement: Translated Abstracts [file supp_czu010_czu010_French.pdf]

Définir les priorités en matière de services de santé dans les hôpitaux : bilan d'études empiriques

Accepté le 30 janvier

La définition des priorités s'est concentrée sur le niveau macro (national) et micro (au chevet du malade), délaissant le niveau institutionnel. Ceci a de quoi surprendre étant donné le rôle clé que les hôpitaux jouent pour les prestations de services et la large part des ressources absorbées par ces derniers. Afin d'étudier les facteurs qui ont un impact sur la définition de ces priorités au niveau d'un hôpital, nous avons mené un bilan thématique d'études empiriques. La recherche systématique des bases de données PubMed, EBSCOHOST, Econlit et google scholar a été complétée par la recherche sur des sites web spécialisés et par la recherche de documents pertinents. Un total de 24 documents a pu être identifié dans les pays développés et en développement. Nous avons appliqué un cadre d'étude des mesures prises pour analyser et synthétiser les résultats des documents sélectionnés. Les résultats montrent que les priorités en matière de services de santé dans les hôpitaux sont influencées par (1) les facteurs contextuels tels que le temps de décision, la disponibilité des ressources, les arrangements financiers, la disponibilité et l'utilisation d'information, la culture organisationnelle et le management, (2) les processus de définition des priorités dépendent du type d'activités, (3) les facteurs de contenu tels que la priorité selon les critères et (4) les protagonistes, leurs intérêts et leurs relations. Nous pouvons dire qu'il y a un besoin certain d'étudier plus en profondeur ces problématiques ainsi que les interactions entre ces études et également d'étudier le cadre conceptuel proposé qui peut être utile pour définir les priorités en matière de services de santé dans les hôpitaux.

Mots clés : définition des priorités, rationnement des soins, planification des soins, hôpitaux
