# Supplementary material for: Setting healthcare priorities in hospitals: a review of empirical studies
Source: Health Policy Plan. 2014 Mar 5;30(3):386–96. doi: 10.1093/heapol/czu010 (PMC4353893; doi:10.1093/heapol/czu010)
Supplement: Translated Abstracts [file supp_czu010_czu010_Spanish.pdf]

El establecimiento de prioridades de salud en los hospitales: una revisión de estudios empíricos

Edwine W. Barasa, Sassy Molyneux, Mike English y Susan Cleary

Aceptado 30 de enero de 2014

El establecimiento de prioridades de investigación se ha centrado en el nivel macro (nacional) y en el micro (al lado de la cama), dejando el nivel meso (institucional, hospital) relativamente descuidado. Esto es sorprendente, debido al papel fundamental que los hospitales desempeñan en la prestación de servicios de salud y la gran proporción de los recursos del sistema de salud que estos absorben. Para explorar los factores que tienen impacto en el establecimiento de prioridades a nivel hospitalario, se realizó una revisión temática de los estudios empíricos. Una búsqueda sistemática en PubMed, EBSCOHOST, bases de datos EconLit y Google scholar se complementó con una búsqueda de sitios web claves y una búsqueda manual de las listas de referencia de papeles pertinentes. Se identificó un total de 24 artículos procedentes de países desarrollados y en vía de desarrollo. Aplicamos un marco de análisis de políticas para examinar y sintetizar los resultados de los trabajos seleccionados. Los resultados sugieren que la práctica en el establecimiento de prioridades en los hospitales fue influenciada por (1) factores contextuales, tales como espacio de decisión, disponibilidad de recursos, arreglos de financiación, disponibilidad y uso de la información, cultura organizacional y liderazgo, (2) los procesos de establecimiento de prioridades que dependen del tipo de actividad de establecimiento de prioridades, (3) los factores de contenido, tales como los criterios utilizados para fijar prioridades y (4) los actores, sus intereses y relaciones de poder. Observamos que existe la necesidad de realizar estudios para examinar estas cuestiones y la interacción entre ellas con mayor profundidad y proponer un marco conceptual que podría ser útil en la evaluación de las prácticas de establecimiento de prioridades en los hospitales.

Palabras Claves
Establecimiento de prioridades, racionamiento del cuidado de la salud, planificación del cuidado de la salud, hospitales

MENSAJES CLAVES

- Hay una escasez de trabajo empírico sobre las prácticas de establecimiento de prioridades a nivel de hospitales y aún más en hospitales rurales más pequeños en el contexto de países en vía de desarrollo.
- La mayoría de los trabajos empíricos identificados se centran en el establecimiento de prioridades en hospitales de mayor tamaño, a menudo hospitales de referencia en países desarrollados.
- Los factores en juego en las prácticas de establecimiento de prioridades del hospital incluyen (1) los factores contextuales, tales como espacio de decisión, disponibilidad de recursos, arreglos de financiación, disponibilidad y uso de la información, cultura organizacional y liderazgo, (2) los procesos de establecimiento de prioridades, (3) los factores de contenido, como por ejemplo, los criterios utilizados para fijar prioridades y (4) los actores, sus intereses y relaciones de poder.
- Una investigación que tenga como objetivo examinar las prácticas en el establecimiento de prioridades en los hospitales se beneficiaría de la aplicación de un enfoque de política de salud a su análisis.
